# Nonlinear T-Wave Time Warping-Based Sensing Model for Non-Invasive Personalised Blood Potassium Monitoring in Hemodialysis Patients: A Pilot Study

**DOI:** 10.3390/s21082710

**Published:** 2021-04-12

**Authors:** Flavio Palmieri, Pedro Gomis, José Esteban Ruiz, Dina Ferreira, Alba Martín-Yebra, Esther Pueyo, Juan Pablo Martínez, Julia Ramírez, Pablo Laguna

**Affiliations:** 1 Centre de Recerca en Enginyeria Biomèdica, Universitat Politècnica de Catalunya, 08028 Barcelona, Spain; flavio.palmieri@upc.edu (F.P.); pedro.gomis@upc.edu (P.G.); 2 CIBER en Bioingeniería, Biomateriales y Nanomedicina (CIBER-BBN), 50018 Zaragoza, Spain; amartiny@unizar.es (A.M.-Y.); epueyo@unizar.es (E.P.); jpmart@unizar.es (J.P.M.); 3 Laboratorios Rubió, Castellbisbal, 08755 Barcelona, Spain; dferreira@labrubio.com; 4 Escuela Superior de Ingeniería, Ciencia y Tecnología, Universidad Internacional de Valencia, 46002 Valencia, Spain; 5 Nephrology Ward, Hospital Clínico Universitario Lozano Blesa, 50009 Zaragoza, Spain; jeruizl@salud.aragon.es; 6 BSICoS Group, I3A, IIS Aragón, Universidad de Zaragoza, 50018 Zaragoza, Spain; 7 William Harvey Research Institute, Queen Mary University of London, London E1 4NS, UK; j.ramirez@qmul.ac.uk

**Keywords:** electrocardiogram, periodic component analysis, T-wave morphology, time warping, noninvasive potassium sensing, personalised medicine

## Abstract

Background: End-stage renal disease patients undergoing hemodialysis (ESRD-HD) therapy are highly susceptible to malignant ventricular arrhythmias caused by undetected potassium concentration ([$K^{+}$]) variations ($\Delta [K^{+}]$) out of normal ranges. Therefore, a reliable method for continuous, noninvasive monitoring of [$K^{+}$] is crucial. The morphology of the T-wave in the electrocardiogram (ECG) reflects $\Delta [K^{+}]$ and two time-warping-based T-wave morphological parameters, $d_{w}$ and its heart-rate corrected version $d_{w,c}$, have been shown to reliably track $\Delta [K^{+}]$ from the ECG. The aim of this study is to derive polynomial models relating $d_{w}$ and $d_{w,c}$ with $\Delta [K^{+}]$, and to test their ability to reliably sense and quantify $\Delta [K^{+}]$ values. Methods: 48-hour Holter ECGs and [$K^{+}$] values from six blood samples were collected from 29 ESRD-HD patients. For every patient, $d_{w}$ and $d_{w,c}$ were computed, and linear, quadratic, and cubic fitting models were derived from them. Then, Spearman’s (ρ) and Pearson’s (*r*) correlation coefficients, and the estimation error ($e_{d}$) between $\Delta [K^{+}]$ and the corresponding model-estimated values ($\hat{\Delta }\left[K^{+}\right]$) were calculated. Results and Discussions: Nonlinear models were the most suitable for $\Delta [K^{+}]$ estimation, rendering higher Pearson’s correlation (median 0.77 ≤r≤ 0.92) and smaller estimation error (median 0.20 $\leq e_{d}\leq$ 0.43) than the linear model (median 0.76 ≤r≤ 0.86 and 0.30 $\leq e_{d}\leq$ 0.40), even if similar Spearman’s ρ were found across models (median 0.77 ≤ρ≤ 0.83). Conclusion: Results support the use of nonlinear T-wave-based models as $\Delta [K^{+}]$ sensors in ESRD-HD patients.

## 1. Introduction

Heart failure is among the most common cardiovascular complications in end-stage renal disease (ESRD) patients [1,2]. In hemodialysis (HD)-dependent ESRD (ESRD-HD) patients, the risk of cardiovascular mortality caused by electrical instability is 10- to 20-fold higher than in age- and gender-matched healthy subjects [3,4]. This remarkable association can be explained by the extreme fluctuations in blood potassium concentration ([$K^{+}$]) occurring in between HD sessions [5,6]. These changes in [$K^{+}$] are usually clinically silent and occur without warning to the patient or to the doctor in the absence of blood tests [7]. Therefore, continuous noninvasive monitoring of [$K^{+}$] variations ($\Delta [K^{+}]$) is of great importance [8] as it would provide risk warnings, improving an ever-growing clinical need.

The electrocardiogram (ECG) reflects the electrical activity of the heart in a non-invasive and inexpensive way. Electrocardiographic consequences of $\Delta [K^{+}]$ are well known [9,10,11]: The earliest effects appear as narrowed and peaked T waves [12], followed by changes in the QT interval duration (or its corrected version, QTc) [13] and in repolarisation complexity [14]. Several studies in the literature have attempted to estimate [$K^{+}$] through the analysis of T-wave morphology changes, quantified by features representative of the T-wave shapes, [15,16,17]. In previous studies [18,19,20,21,22], we proposed and investigated six T-wave morphological parameters quantifying T-wave morphology changes by means of time warping analysis [23] for continuous non-invasive $\Delta [K^{+}]$ monitoring. These six T-wave morphology parameters included $d_{w}^{u}$, $d_{w}$, and ${\hat{d}}_{w,c}$ (unsigned, signed, and heart rate corrected T-wave morphology variations in time, respectively), $d_{a}$ (T-wave morphology variations in amplitude), and their non-linear components ($d_{w}^{\mathrm{NL}}$ and $d_{a}^{\mathrm{NL}}$) as described in [21,23]. In addition, we tested two lead space reduction techniques [20], principal component analysis (PCA) and periodic component analysis (πCA) [24], this later implemented in two different versions: By exploiting the complete QRST complex periodicity, $\pi C^{B}$, or just restricting to the T-wave, $\pi C^{T}$ [20]. This work [20] showed that $d_{w}$ and ${\hat{d}}_{w,c}$ had the highest correlation with $\Delta [K^{+}]$. They also showed that $\pi C^{T}$ presented higher robustness against noise than PCA or $\pi C^{B}$, making it the most suitable lead space reduction technique for $\Delta [K^{+}]$ tracking during the HD session, as well as in the post therapy monitoring before the next HD session. Nevertheless, a quantitative relation between these T-wave morphological parameters derived from ECG analysis and $\Delta [K^{+}]$ has not yet been established for clinical use, which would allow a non invasive measurement of $\Delta [K^{+}]$ value.

**Figure 1:**
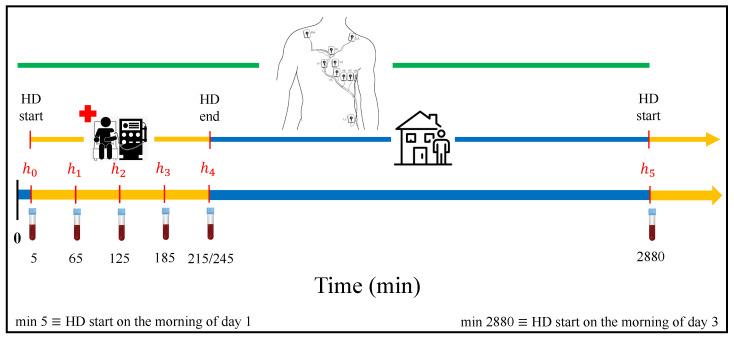
Data acquisition protocol. Holter electrocardiogram (ECG) signals of end-stage renal disease patients undergoing hemodialysis (ESRD-HD) patients were acquired throughout 48 h, starting 5 min before the beginning of the HD therapy. Six blood samples were collected at the beginning of the therapy ($h_{0}$), each hour during the HD ($h_{1}$, $h_{2}$, $h_{3}$), at the end ($h_{4}$, at minute 215th or 245th, depending on the HD duration) and before the beginning of the next HD session ($h_{5}$).

**Figure 2:**
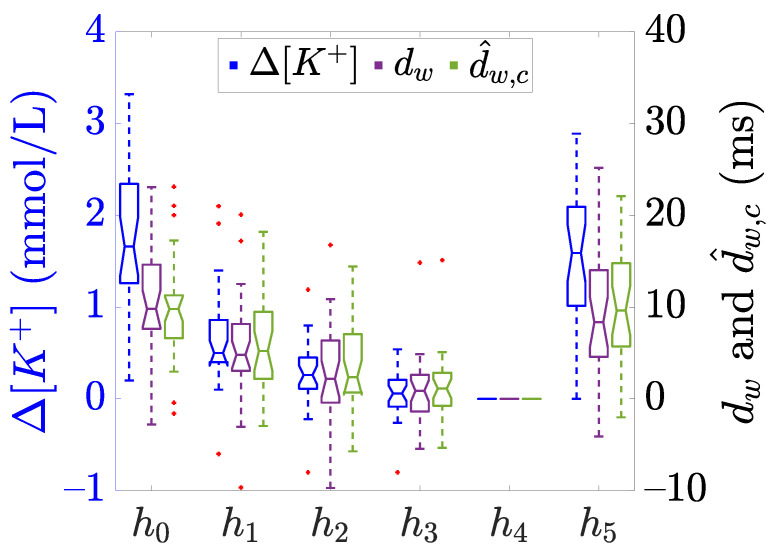
Boxplots showing the distribution of $\Delta [K^{+}]$ (blue) and the described $\pi C^{T}$-based time warping biomarkers $d_{w}$ (purple) and ${\hat{d}}_{w,c}$ (green), computed at each time points ($h_{0}$ to $h_{5}$), see Figure 1. The central line of the boxplots represents the median, the edges of the box are the 25-th and 75-th percentiles, and the whiskers extend to the most extreme data points not considered as outliers. The notches represent the 95% confidence interval of the median, calculated as $q_{2}-1.57\left(q_{3}-q_{1}\right)/\sqrt{n}$ and $q_{2}$ + $1.57\left(q_{3}-q_{1}\right)/\sqrt{n}$ being $q_{2}$ the median, $q_{1}$ and $q_{3}$ are the 25-th and 75-th percentiles, respectively, and *n* is the sample size. Finally, red “+” denotes outliers. Data adapted from [20,21].

The direct assessment of a marker as $\left[K^{+}\right]$ surrogate by Pearson correlation analysis implies the assumption of a linear relation between them. However, previous works have reported that the reconstruction of [$K^{+}$] from the ECG significantly improves by employing a quadratic regression [16]. This result is compatible with the findings we reported in [20,21] where the same study population described in Section 2 was investigated according to the protocol in Figure 1. In Palmieri et al. [21], we observed a non-linear correspondence between $\Delta [K^{+}]$ and the T-wave time warping biomarkers $d_{w}$ and ${\hat{d}}_{w,c}$ (purple and green boxplots, respectively, in Figure 2 of the present study). Therefore, we hypothesised that using patient-specific nonlinear models based on T-wave time warping-derived markers can provide better quantitative assessment of $\Delta [K^{+}]$. The aim of this study is to derive and to evaluate nonlinear polynomial sensing models to estimate $\Delta [K^{+}]$ by using $\pi C^{T}$-based markers, $d_{w}$ and ${\hat{d}}_{w,c}$. As a reference, a patient-specific linear model is also estimated for each marker.

This paper is organised as follows. First, we describe the study population, a database recorded during the interdialytic interval between two HD sessions. We next expand on the methodology to calculate the T-wave morphology parameters, as well as the proposed models to monitor [$K^{+}$] fluctuations from the ECG. Finally, we present and discuss the results, ending with conclusions and considerations for future research.

## 2. Materials

The ESRD-HD study population included 29 patients from the Nephrology ward at Hospital Clínico Universitario Lozano Blesa (Zaragoza, Spain). Inclusion criteria were (i) 18-year-old or older patients, (ii) being diagnosed with ESRD, and (iii) undergoing HD at least three times per week, with venous or cannula access. The study protocol was approved by the Aragon’s ethics committee (CEICA, ref. PI18/003) and all patients signed informed consent. All procedures and methods were performed in accordance with the Helsinki Declaration. Further details concerning the study protocol and clinical features of the study population can be found in [20,21]. A 48-h, standard 12-lead ECG Holter recording (H12+, Mortara Instruments, Milwaukee, WI, USA, sampling frequency of 1 kHz, amplitude resolution of 3.75 μV) was collected for each enrolled patient, with the acquisition starting 5 min before the HD onset and lasting until the next HD session, programmed 48 h later. Simultaneously, to determine [$K^{+}$], a total of 6 blood samples were collected just before starting the HD and every hour during the HD session (5 in total), with a last extraction immediately before the next HD session (Figure 1). The current number of patients included in the database is still limited, hence this work should be interpreted as an exploratory pilot study.

## 3. Methods

**Figure 3:**
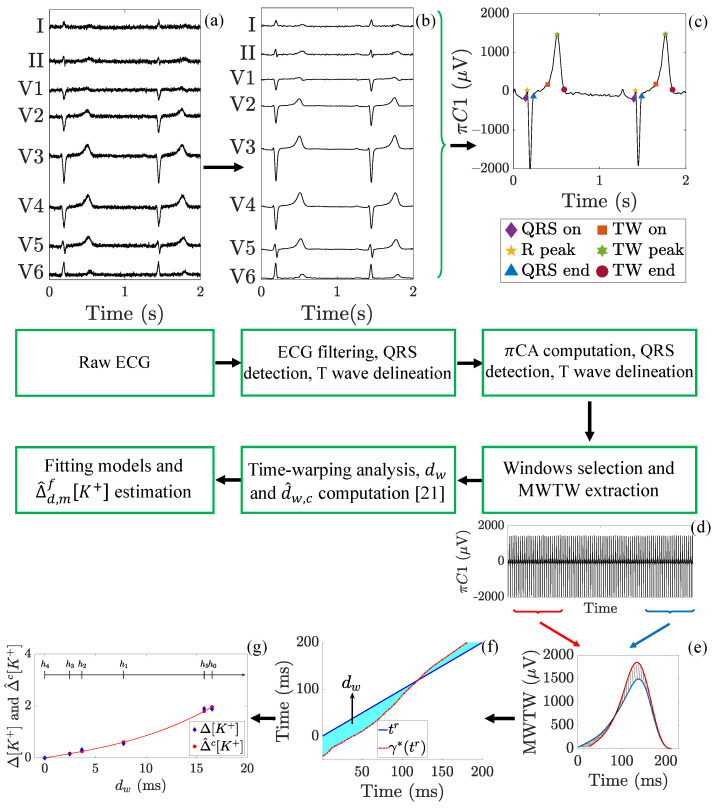
Flow chart showing the ECG processing steps performed in this study. (**a**) Raw ECG (the eight independent leads I, II, V1 to V6 are shown) obtained from one of the enrolled ESRD-HD patients (see Section 2). (**b**) Preprocessed ECG as described in Section 3.1. (**c**) πCA is applied and both QRS complexes and T-waves (TW in the legend) are detected and delineated as detailed in Section 3.2. (**d**) From 2-min wide windows, (**e**) a mean warped T-wave (MWTW) is extracted and (**f**) T-wave morphology markers $d_{w}$ and ${\hat{d}}_{w,c}$ are computed as stated in Section 3.3. (**g**) The fitting models for ${\hat{\Delta }}_{d,m}^{f}\left[K^{+}\right]$ estimation are evaluated as in Section 3.5. In this example, a cubic model with *m* = *a* is presented.

In this section, the different steps required for the processing of the ECG signals are described and summarised in the block diagram presented in Figure 3.

### 3.1. ECG Pre-Processing

Baseline wander was removed with a 0.5-Hz cut-off high-pass filter, implemented with a forward-backward 6-th order Butterworth filter [25]. Residual noise out of the T-wave band was removed with a 40 Hz cut-off frequency forward-backward 3-th order low-pass Butterworth filter. QRS complexes were detected and T-waves delineated using a wavelet-based single-lead delineation method applied to each of the 12 leads [26].

### 3.2. Lead Transformation by Periodic Component Analysis, πCA

Periodic component analysis is a lead space reduction technique aiming to emphasise the periodic structure of a signal [24,27]. In this work, πCA was applied with a one-beat periodicity to maximise the T-wave beat-to-beat periodic components on the transformed signal, as explained in [20]. For each ECG recording, a transformation matrix $\Psi_{\pi \mathrm{CA}}$ was estimated as detailed in [20], and applied to the 8 independent standard leads, obtaining a new set of 8 transformed leads, named periodic components. In this way and by ordering the transformed leads inversely to their associated eigenvalue, the most beat-to-beat periodic components appear projected onto the first component, πC1, which was selected for subsequent analysis and T waves were again delineated by using the above-mentioned delineator [26].

### 3.3. Warping-Based T-Wave Morphology Markers

All T-waves from πC1 were further low-pass filtered at 20 Hz using a forward-backward 6-th Butterworth filter to remove remaining out-of-band frequency components. T-waves in 2-min wide windows centered around the 5-th minute and 35-th minute of each available hour were selected, from which a mean warped T-wave (MWTW) was computed from all T-waves in each window [21,23]. Finally, the two T-wave morphology parameters, $d_{w}$ and $d_{w,c}$, were computed by comparing each MWTW with respect to a reference MWTW, selected at the end of the HD session, resulting in relative markers to the reference point at the end of HD ($h_{4}$ in Figure 1). A detailed description of the computation of the warping markers here analysed can be found in [21,23], describing how $d_{w}$ represents a relative measure of morphological changes between two T-waves. Likewise, ${\hat{d}}_{w,c}$ is obtained from $d_{w}$ marker after being compensated for T-wave morphological changes not attributable to $\Delta [K^{+}]$ but to heart rate changes occurring between the reference and analysis points [20,21].

### 3.4. Blood Potassium Concentration Variations $\Delta [K^{+}]$

The two proposed biomarkers, measured along time, have been associated with the corresponding relative variations in [$K^{+}$] with respect to the [$K^{+}$] at the reference point ($h_{4}$), where a blood sample was taken:

(1) $$\Delta \left[K^{+}\right]\left(h_{i}\right)={\left[K^{+}\right]}_{h_{i}}-{\left[K^{+}\right]}_{h_{4}}$$

being ${\left[K^{+}\right]}_{h_{i}}$ the concentration at the $h_{i}$-th time point (see Figure 1) and ${\left[K^{+}\right]}_{h_{4}}$ the reference concentration at the end of the HD treatment. The $\Delta [K^{+}]$ distribution across patients for each hour is presented in Figure 2.

### 3.5. Marker Fitting Models for $\Delta [K^{+}]$ Estimation

For a given patient *p*, the relationship between the marker $d\in \{d_{w},d_{w,c}\}$ and $\Delta [K^{+}]$ measured along time was modelled by means of a linear (*l*), quadratic (*q*), and cubic (*c*) regression models for each patient to noninvasively calculate $\Delta [K^{+}]$ values, according to the following models:

(2) $${\hat{\Delta }}_{d}^{l}\left[K^{+}\right]\left(h_{i}\right)=\alpha_{l}\;d\left(h_{i}\right),$$

(3) $${\hat{\Delta }}_{d}^{q}\left[K^{+}\right]\left(h_{i}\right)=\alpha_{q}\;d\left(h_{i}\right)+\beta_{q}\;d^{2}\left(h_{i}\right),$$

(4) $${\hat{\Delta }}_{d}^{c}\left[K^{+}\right]\left(h_{i}\right)=\alpha_{c}\;d\left(h_{i}\right)+\beta_{c}\;d^{2}\left(h_{i}\right)+\gamma_{c}\;d^{3}\left(h_{i}\right),$$

respectively. The coefficients $\alpha_{l}$, $\alpha_{q}$, $\beta_{q}$, $\alpha_{c}$, $\beta_{c}$, and $\gamma_{c}$ were estimated for each patient *p* and marker *d* by using a least square regression analysis between $\hat{\Delta }\left[K^{+}\right]$ and $\Delta [K^{+}]$ values. For each patient and marker, the parameters of the three models were estimated with two different approaches: (i) By using all the available $\Delta [K^{+}]$ values (“*m* = *a*”) and (ii) by adopting a leave-one-out cross validation (“*m* = *o*”) by excluding the $h_{i}$-th $\Delta \left[K^{+}\right]\left(h_{i}\right)$ value from the training-set and evaluating the prediction error at this $h_{i}$-th point, repeating this for all possible $h_{i}$ exclusions.

**Figure 4:**
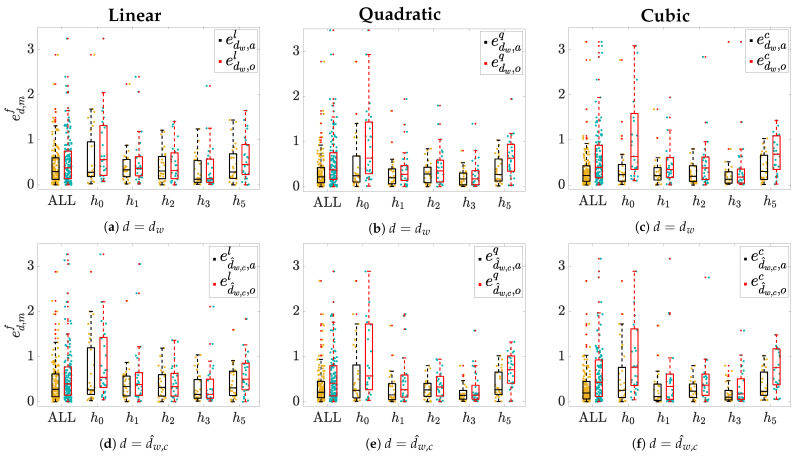
Estimation error ($e_{d,m}^{f}\left(p,h_{i}\right)$) distributions across patients for each hour *h_i_* and when pooling all samples together (ALL). Panels (**a**,**d**) show results for linear models *f* = *l*; panels (**b**,**e**) show the quadratic and panels *f* = *q*; and (**c**,**f**) show the cubic model *f* = *c*. Yellow dots represent individual error values when m = a, while light-blue ones denote those obtained when *m* = *o*. Corresponding boxplots are depicted on top of each distribution: The black ones represent the errors in *m* = *a* while the red ones represents error in case of *m* = *o*. “+” denotes outliers.

Finally, to avoid physiologically meaningless ${\hat{\Delta }}_{d}\left[K^{+}\right]$ trends, the three models in Equations (2)–(4) were computed with a constrained parameter estimation in order to guarantee a monotonically increasing relationship between $\hat{\Delta }\left[K^{+}\right]$ and *d*, as physiologically expected and corroborated by the marker trend evolution in Figure 2 in this paper and in Corsi et al. [16] in Figure 2 and Figure 4. That was implemented by imposing:

(5) $$\frac{\partial {\hat{\Delta }}_{d}\left[K^{+}\right]}{\partial d}\geq 0,$$

which for positive values of the marker, d>0, implies $\alpha_{l}\geq 0$, $\alpha_{q}\geq 0$, $\beta_{q}\geq 0$, $\alpha_{c}\geq 0$, $\beta_{c}\geq 0$, and $\gamma_{c}\geq 0$. The case with d<0 is anecdotal, see Figure 2, and most likely is due to outliers, since they do not follow physiological interpretations of T-wave narrowing with increased potassium.

### 3.6. Statistical Analysis

Spearman’s rank and Pearson’s correlation coefficients (ρ and *r*, respectively) were used for correlation analyses between $\Delta [K^{+}]$ and ${\hat{\Delta }}_{d,m}^{f}\left[K^{+}\right]$, where f∈{l,q,c} denotes the fitting model, $d\in \{d_{w},{\hat{d}}_{w,c}\}$ the T-wave morphology parameter and m∈{a,o} the estimation method. This analysis gives information about both the monotonic relation and the strength of the association between each modelled T-wave morphology parameter and $\Delta [K^{+}]$, thus providing a more complete characterisation. In addition, for each patient *p* and hour $h_{i}$, an estimation error $e_{d,m}^{f}\left(h_{i},p\right)$ was computed as:

(6) $$e_{d,m}^{f}\left(h_{i},p\right)=\left|{\hat{\Delta }}_{d,m}^{f}\left[K^{+}\right]\left(h_{i},p\right)-\Delta \left[K^{+}\right]\left(h_{i},p\right)\right|$$

where i∈{0,1,2,3,5} is the set of hours where the computation of the estimation error is meaningful. Note that $h_{4}$ is the reference point where both $\Delta [K^{+}]$ and ${\hat{\Delta }}_{d,m}^{f}\left[K^{+}\right]$ are equal to zero and therefore it is excluded from error computation to avoid a biased error evaluation. The value ${\hat{\Delta }}_{d,a}^{f}\left[K^{+}\right]\left(p,h_{i}\right)$ represents the estimation at time $h_{i}$ when training is done including all available $h_{i}$ hours values from the corresponding patient, while ${\hat{\Delta }}_{d,o}^{f}\left[K^{+}\right]\left(p,h_{i}\right)$ represents the estimate at hour $h_{i}$ when all but the $h_{i}$-th point of the patient are used in the training.

All statistical analyses were performed using MATLAB version R2019a and results are given as the median and interquartile range (IQR).

## 4. Results

The median and IQR values of intra-patient Spearman’s (ρ) and Pearson’s (*r*) correlation coefficients, computed between $\Delta [K^{+}]$, and ${\hat{\Delta }}_{d,m}^{f}\left[K^{+}\right]$, are given in Table 1. The same table also displays the median and IQR values of the errors $e_{d,m}^{f}\left(p,h_{i}\right)$, pooling together all patients and all blood extractions (ALL), and segregated for hour $h_{0}$ and $h_{5}$.

Boxplots in Figure 4 show the estimation error $e_{d,m}^{f}\left(p,h_{i}\right)$ distributions, sorted by hours $h_{i}$, using the linear (Figure 4a,d), the quadratic (Figure 4b,e), and the cubic (Figure 4c,f) models. In addition, the aggregated distribution for all hours is presented with the label (ALL). The widest error distributions are obtained for hours $h_{0}$ and $h_{5}$, whose median and IQR are given in Table 1. These time points are of great interest since: (i) The samples are the furthest from the reference ($h_{4}$) and (ii) when they are estimated by using the leave-one-out (*m* = *o*) approach they do not have any temporarily close samples before (i.e., in case of $h_{0}$) and/or after (i.e., in case of $h_{5}$) as opposed to $h_{1}$, $h_{2}$, and $h_{3}$; this together with the fact that their associated marker values are also the farthest from the rest, Figure 2. Therefore, it seemed worthy performing a detailed hour-based error analysis.

**Figure 5:**
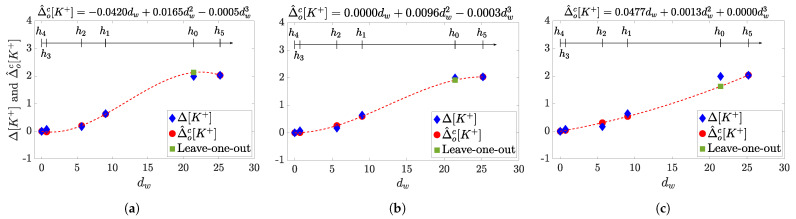
Examples of cubic models (red dotted lines) computed for a given patient by imposing different parameter restrictions for leave-on-out cross-validation method, the corresponding equations are reported above each panel. The resulting model without restrictions on $\left\{\alpha_{c},\beta_{c},\gamma_{c}\right\}$ is in panel (**a**), while those from imposing *α_c_* ≥ 0, or full constrained model are presented in (**b**,**c**) respectively. In each panel: The blue diamonds represent measured $\Delta [K^{+}]$ values at the hours $\left\{h_{0},h_{1},h_{2},h_{3},h_{4},h_{5}\right\}$; while red dots are the estimated ${\hat{\Delta }}_{d_{w},o}^{c}\left[K^{+}\right]$ corresponding to the computed *d_w_* used in the training set and computed at $\left\{h_{1},h_{2},h_{3},h_{4},h_{5}\right\}$; the green square is the estimated ${\hat{\Delta }}_{d_{w},o}^{c}\left[K^{+}\right]$ corresponding to the *d_w_* at *h_0_*, the hour excluded from the training set in this example, and then the one with higher risk for error in the estimation. See that only full set of parameters forced to be positive result in a monotonic, physiologically plausible, function.

An example of cubic modeling results for a given patient with and without parameter constriction for monotonic ${\hat{\Delta }}_{d_{w},o}^{c}\left[K^{+}\right]$ behaviour with *d* is presented in Figure 5. Results are given for no restrictions on $\left\{\alpha_{c},\beta_{c},\gamma_{c}\right\}$ (Figure 5a); by just imposing $\alpha_{c}\geq 0$ (Figure 5b); and by the full constrained model ($\alpha_{c}\geq 0$, $\beta_{c}\geq 0$, $\gamma_{c}\geq 0$) (Figure 5c).

## 5. Discussion

In this study, we analysed ECG signals from 29 ESRD-HD patients. We extracted T-wave morphology parameters, $d_{w}$ and ${\hat{d}}_{w,c}$, previously reported to have a strong correlation with $\Delta [K^{+}]$ [20,21]. Then, we proposed and compared, the use of linear, quadratic, and cubic regression models for $\Delta [K^{+}]$ estimation from $d_{w}$ and ${\hat{d}}_{w,c}$ markers. The performance of each model was evaluated through Spearman’s and Pearson’s correlation coefficients of the estimated $\hat{\Delta }\left[K^{+}\right]$ with respect to actual $\Delta [K^{+}]$ values and through hourly-based absolute estimation errors. The results on ESRD-HD patients here reported showed that non-linear regression models could be advantageously used to quantitatively estimate $\Delta [K^{+}]$ and could, therefore, be an effective tool for remote, frequent, and noninvasive monitoring of ESRD-HD patients.

According to Spearman’s correlation coefficient (ρ) between measured and estimated variations in [$K^{+}$], similar ρ median values were found across the three models, with 0.06 being the highest median increment when moving from a linear to a cubic model for $d=d_{w}$ in *m* = *a* and, thus, denoting an analogous monotonic relationship between real $\Delta [K^{+}]$ and estimated values (${\hat{\Delta }}_{d,m}^{f}\left[K^{+}\right]$). On the other hand, an improvement can be appreciated when comparing Pearson’s correlation coefficient (*r*) evaluated in the three models, being the IQR reduced in $d=d_{w}$ and *m* = *a* by 0.06 and 0.08 when comparing the quadratic and cubic models, respectively, with respect to the linear model. Similar considerations can be made for $d={\hat{d}}_{w,c}$. This is an expected outcome since the models here proposed were designed to avoid distorting the original monotonic increasing relationship between $\Delta [K^{+}]$ and the ECG derived markers, but only to adjust for the linear/non-linear relationship between them. However, the overall performance decreases considerably when the leave-one-out method, *m* = *o*, was used, being the median *r* lower and the IQR wider than in *m* = *a*. Also, for both $d_{w}$ and ${\hat{d}}_{w,c}$ in *m* = *o*, a remarkable increase in the IQR can be observed when comparing linear and cubic models: From 0.47 to 0.61 for the first marker and from 0.34 to 0.45 for the second one. Overall, these findings seem to suggest that the cubic model does not provide any additional advantages to the linear or quadratic models in estimating $\Delta [K^{+}]$ using the leave-one-out approach. Therefore, the results we observed for *m* = *a* could potentially be affected by over-fitting.

Another interesting observation can be made when comparing $d_{w}$ with ${\hat{d}}_{w,c}$ in terms of *r*: For the linear model and *m* = *a*, a small gain is obtained by heart rate correction, which is more significant for *m* = *o*. However, this improvement for the heart rate corrected index ${\hat{d}}_{w,c}$ vanishes in *m* = *a* when using the quadratic model or the cubic model getting even worse in *m* = *o*. This can also be a result of the over-fitting in these estimates, ${\hat{d}}_{w,c}$ since it is already subjected to an heart rate correction estimation [21].

A reduction in the median and IQR estimation error for $d=d_{w}$ in *m* = *a* results when hours and patients values are pooled together. The IQR decreases from 0.48 for the linear model to 0.34 for both the quadratic and cubic models. The median error goes from 0.30 in the linear model to 0.22 and 0.21 in the quadratic and the cubic models, respectively. An analogous trend can be found for $d={\hat{d}}_{w,c}$ in *m* = *a*: IQR reduces from 0.50 in f=l to 0.36 in f=q and to 0.39 in f=c. However, for both markers, the improvements disappear when the leave-one-out method *m* = *o* is used, which would support the previously hypothesised over-fitting for *m* = *a*. These outcomes would point at the quadratic model as the most suitable model for $\Delta [K^{+}]$ estimation in *m* = *a*, as well as in *m* = *o*, even if in this latter case the advantage is not very remarkable. Moreover, as mentioned above, there is no clear benefit in using a cubic rather than a quadratic model in any of both *m* = *a* and *m* = *o* cases, probably due to the full constrained parameter estimation rule we imposed which, when applied to the cubic model, we observed it resulted in a very small cubic term, reducing to quadratic model as in Figure 5.

The most distant hours from the reference point ($h_{0}$ and $h_{5}$ in this work) are the most interesting and challenging for $\Delta [K^{+}]$ estimation. Indeed, these two are the time points where the estimation errors are the highest and the error distributions are the widest, which is particularly true when they are taken out of the training set in the *m* = *o* case (red boxplot). That could be considered as an indication of the high uncertainty in predicting such values, especially when the values to be estimated do not have closer samples before and/or after, resulting in the wide IQR values reported for $e_{d,m}^{f}$ at $h_{0}$ and $h_{5}$, Table 1. In general, the IQR value for $e_{d,m}^{f}$ at $h_{0}$ decreases for $d_{w}/{\hat{d}}_{w,c}$ in *m* = *a* from 0.77/1.03 for linear to 0.58/0.73 for quadratic and to 0.37/0.67 for cubic model (similarly for the median), but again these reductions vanish in *m* = *o*. Analogous considerations can be made for $e_{d,m}^{f}$ at $h_{5}$.

**Figure 6:**
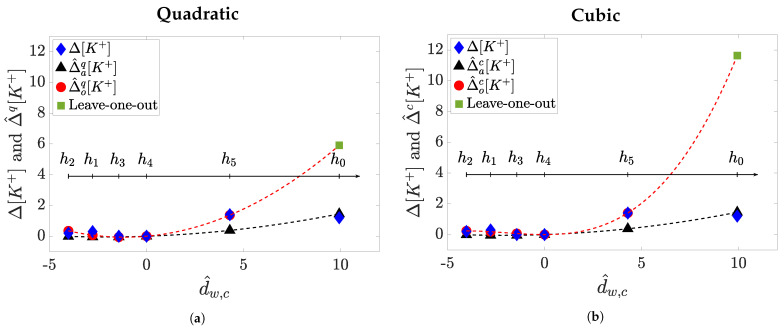
Example of leave-one-out model prediction (*m* = *o*) at *h_0_* compared to a *m* = *a* approach for a given patient. The quadratic models (*f* = *q*) are depicted in panel (**a**) while the cubic ones (*f* = *c*) are in panel (**b**). In each panel: The blue diamonds represent measured $\Delta [K^{+}]$ values at each hour $\left\{h_{0},h_{1},h_{2},h_{3},h_{4},h_{5}\right\}$; the black triangles are the estimated ${\hat{\Delta }}_{{\hat{d}}_{w,c},a}^{f}\left[K^{+}\right]$ while the red dots are ${\hat{\Delta }}_{{\hat{d}}_{w,c},o}^{f}\left[K^{+}\right]$ corresponding to the ${\hat{d}}_{w,c}$ used in the training set $\left\{h_{1},h_{2},h_{3},h_{4},h_{5}\right\}$, and the green square is the predicted ${\hat{\Delta }}_{{\hat{d}}_{w,c},o}^{f}\left[K^{+}\right]$ corresponding to the ${\hat{d}}_{w,c}$ at *h_0_*, the hour excluded from the training set. The blackdashed line is the model in *m* = *a* while the red-dashed line accounts for the model in *m* = *o*.

The results observed so far may lead to the conclusion that, according to the performance metrics *r* or $e_{d,o}^{f}$ considered, the observed improvement for quadratic model estimation in the case of *m* = *a* vanishes, or it is largely attenuated, in *m* = *o*. However, when analysing data distributions we realise that values of $d_{w}$ and ${\hat{d}}_{w,c}$ markers are not evenly distributed in all the analysed range (see Figure 2). This fact can imply an overweight of small *d* values in *m* = *o* modelling, penalising the estimates at $h_{0}$ and $h_{5}$, which present *d* values that might not be well represented in the training set. This could also mean that the leave-one-out cross-validation needs to be cautiously framed when the value of *d* to be estimated is far from those used in the training set range, which in our dataset usually happens at $h_{0}$ and/or at $h_{5}$ as exemplified in Figure 6. In these cases, the estimation error between real $\Delta [K^{+}]$ and ${\hat{\Delta }}_{d,o}^{f}\left[K^{+}\right]$ would be larger than the error with respect to ${\hat{\Delta }}_{d,a}^{f}\left[K^{+}\right]$. This could be due to the fact that when the training set consists of all the available *d* values (i.e., *m* = *a*), thus covering all the whole spanning range for that patient, the estimated coefficients make a proper modeling and $\Delta [K^{+}]$ estimation possible. However, if that range is not well represented (e.g., in *m* = *o* mainly for $h_{0}$ and $h_{5}$), then the estimated coefficients model well the range of low *d* values, but do not model well large *d* values outside that range, thus not being able to provide accurate estimates for high *d* values, resulting in inconsistent models and then in unreliable $\Delta [K^{+}]$ estimation. This circumstance is particularly true for the cubic model rather than for the quadratic one, as a consequence of having an extra parameter to fit, then increasing the possibility of overfitting, obtaining divergent values outside the training range.

In the following, some limitations of our study are acknowledged. If blood samples had been collected more frequently during the early stage of the HD treatment when [$K^{+}$] and, consequently, *d* more rapidly change—covering a broad range of values—then the model training set in *m* = *o* could have better represented all the possible cases of *d* in the quadratic as well as in the cubic model, and then the results could have been more conclusive for the non-linear modelling improvement in predicting [$K^{+}$]. If this refined learning would have been done, or is done in future studies, it will, predictably, result in less error at the extreme times $h_{0}$ and $h_{5}$ of the process, and consequently also in a notably improved performance of the quadratic model both for *m* = *a* and for *m* = *o*.

Another limitation that should be taken into account when interpreting this work’s results is the lack of perfect time synchronisation between the actual $\Delta [K^{+}]$ and the evaluated *d* used for estimation at $h_{5}$. As previously reported in [21], 44 h is the average ECG duration in our database—not 48 h, when the last blood sample is takenl—mainly due to electrode detachment or early battery exhaustion. However, in a recent study [20], we observed a low marker dynamics in the late post-HD treatment. Therefore, with some degree of confidence, we have assumed that the estimation error obtained between $\Delta [K^{+}]$ and ${\hat{\Delta }}_{d,m}^{f}\left[K^{+}\right]$ at $h_{5}$ would be quite similar if the actual value—had the ECG lasted, as planned, for 48 h—had been used for modelling.

Specific aspects of ESRD-HD patients’ clinical status (e.g., possibility of previous infarction not always revealed in clinical history) could have influenced the results, generating the inter-patient variability here observed. In addition, the accuracy of the proposed models in estimating potassium variations for patients other than ESRD-HD remains to be assessed.

Finally, the reduced number of patients and available blood samples for each patient included in this study also represents a limitation to frame the conclusion of the work. Indeed, even if the proposed approach may entail a significant step towards a robust and reliable $\Delta [K^{+}]$ sensing from time-warping based biomarkers, it needs to be validated in larger cohorts before any translation to clinical practice. However, the available data would suggest that a patient-specific quadratic model could estimate $\Delta [K^{+}]$ time trends with better accuracy than a linear-model. Also, in real practice, this method implies the collection of several blood samples, which may result in cumbersome procedures. It remains to be studied to what extent the models learned in one session can be extrapolated for sessions in later days/weeks, reducing the learning to just a single session.

Future studies should be conducted in a larger population including not only ESRD-HD patients but also subjects at risk of [$K^{+}$] imbalance, such as those with diabetes mellitus [28] or severe cardiovascular events like myocardial infarction [29]. In addition, the proposed estimation models should be validated in a follow-up study where the models are learned at the initial HD session and used in later HD sessions to measure $\Delta [K^{+}]$. In such studies the complete learning with *m* = *a* at the initial HD session could be evaluated by its prediction value at subsequent sessions, without any overfitting risk. At this future analysis, we expect that *m* = *a* approach will show better performance, in terms of correlation and estimation error, than the one reported here for the *m* = *o* case, since the models’ coefficients will be estimated over the six $\Delta [K^{+}]$ values (and not just over five as in *m* = *o*), thus covering the full range of *d* values for each patient.

## 6. Conclusions

The present study showed the advantage in using non-linear models in estimating $\Delta [K^{+}]$ measurements in ESRD-HD patients based on T-wave-derived markers. These results suggest a new noninvasive strategy for ECG-based [$K^{+}$] sensing, with large implications for monitoring patients with cardiovascular and renal diseases, providing a meaningful tool for a personalised ambulatory cardiac risk assessment of these patients.

## Figures and Tables

**Table 1:** Intra-patient ρ, *r*, $e_{d,m}^{f}$—either when pooling all patients and blood samples together (ALL) or specifically for $h_{0}$ and $h_{5}$—evaluated between $\Delta [K^{+}]$ and ${\hat{\Delta }}_{d,m}^{f}\left[K^{+}\right]$, expressed as median (interquartile range (IQR)), for each model f∈{l,q,c}, marker $d\in \{d_{w},{\hat{d}}_{w,c}\}$, and estimation rule m∈{a,o}.

*d*	*f*	*m*	ρ	*r*	$e_{d,m}^{f}$		
					ALL	$h_{0}$	$h_{5}$
$d_{w}$	*l*	*a*	0.83 (0.33)	0.86 (0.35)	0.30 (0.48)	0.28 (0.77)	0.29 (0.55)
		*o*	0.77 (0.48)	0.76 (0.47)	0.38 (0.61)	0.56 (1.10)	0.45 (0.66)
	*q*	*a*	0.83 (0.36)	0.91 (0.29)	0.22 (0.34)	0.24 (0.58)	0.27 (0.49)
		*o*	0.83 (0.49)	0.77 (0.51)	0.38 (0.59)	0.64 (1.15)	0.63 (0.60)
	*c*	*a*	0.89 (0.35)	0.92 (0.27)	0.21 (0.34)	0.23 (0.37)	0.30 (0.54)
		*o*	0.83 (0.49)	0.79 (0.61)	0.39 (0.72)	0.64 (1.24)	0.69 (0.75)
${\hat{d}}_{w,c}$	*l*	*a*	0.83 (0.31)	0.88 (0.34)	0.27 (0.50)	0.26 (1.03)	0.31 (0.54)
		*o*	0.80 (0.44)	0.81 (0.34)	0.40 (0.63)	0.54 (1.11)	0.50 (0.59)
	*q*	*a*	0.83 (0.35)	0.90 (0.27)	0.21 (0.36)	0.25 (0.73)	0.27 (0.50)
		*o*	0.80 (0.53)	0.77 (0.39)	0.41 (0.67)	0.57 (1.45)	0.71 (0.61)
	*c*	*a*	0.83 (0.31)	0.90 (0.25)	0.20 (0.39)	0.25 (0.67)	0.23 (0.52)
		*o*	0.80 (0.49)	0.72 (0.45)	0.43 (0.81)	0.77 (1.25)	0.76 (0.80)

## Data Availability

The dataset is still ongoing and it is available upon request to the corresponding author.

## Abbreviations

The following abbreviations are used in this manuscript:

$\Psi_{\pi \mathrm{CA}}$	Transformation matrix to perform Periodic Component Analysis
$\left[K^{+}\right]$	Blood potassium concentration
$\Delta [K^{+}]$	Blood potassium concentration variations
ECG	Electrocardiogram
ESRD	End Stage Renal Disease
ESRD-HD patients	End Stage Renal Disease patients undergoing hemodialysis
HD	Hemodialysis
IQR	Interquartile range
MWTW	Mean Warped T-wave
PCA	Principal Component Analysis
π CA	Periodic Component Analysis
$\pi C^{T}$	Periodic Component Analysis evaluated over the T-wave
ρ	Spearman’s correlation coefficient
*r*	Pearson’s correlation coefficient
